# Cathepsin L inhibition prevents the cleavage of multiple nuclear proteins upon lysis of quiescent human cells

**DOI:** 10.17912/micropub.biology.000716

**Published:** 2022-12-14

**Authors:** Prashant Gaikwad, Michael G. Kemp

**Affiliations:** 1 Department of Pharmcology and Toxicology, Wright State University Boonshoft School of Medicine, Dayton, OH 45435; 2 Research Service, Dayton VA Medical Center, Dayton, OH 45428

## Abstract

Several studies have indicated a role for cathepsin L (CTSL) proteolytic activity in the nucleus under distinct cellular conditions, including during differentiation, senescence, and quiescence. Here we show that addition of CTSL inhibitors to a cell lysis buffer prevents the cleavage of several nuclear proteins during the lysis of quiescent human cells, including proteins previously thought to have functional relevance in other cell and tissue contexts. These findings suggest that care should be taken to use CTSL inhibitors when lysing cells and tissues containing high levels of CTSL protein to differentiate proteolysis that occurs in vivo versus artifactually in vitro.

**Figure 1. Multiple nuclear proteins are cleaved by cathepsin L (CTSL) when quiescent cells are lysed in the absence of a CTSL inhibitor.**
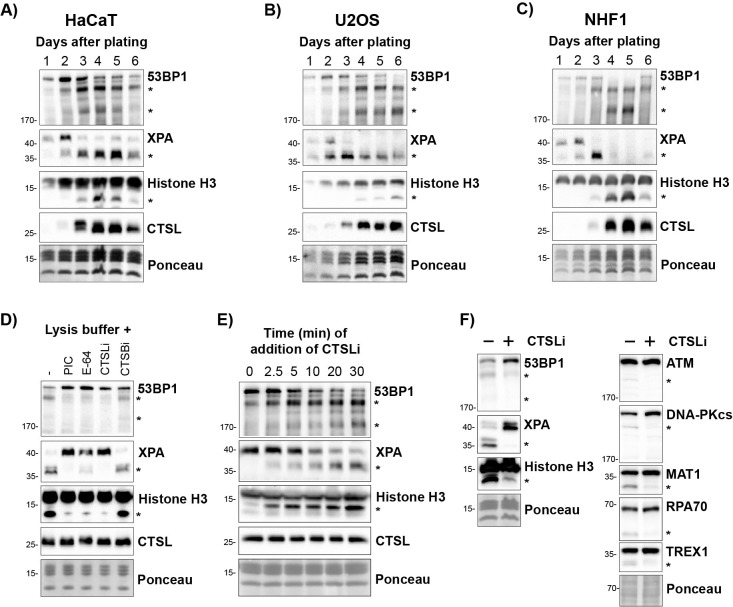
**A)** Protein immunoblotting was performed using lysates prepared from HaCaT cells on the indicated days after plating. **B)** Lysates were prepared from U2OS cells as in A. **C)** Lysates were prepared from NHF1 cells as in A. **D)** Quiescent HaCaT cells were lysed in the absence or presence of a general protease inhibitor cocktail (PIC), the general cysteine protease inhibitor E-64, or specific cathepsin L and B inhibitors (CTSLi/CTSBi). **E)** The CTSL inhibitor was added to the cell lysates at the indicated time points after resuspending quiescent cells in lysis buffer. **F)** Lysates from quiescent HaCaT cells were lysed in buffer lacking or containing the specific CTSLi Z-FY-CHO. Asterisks (*) indicate immunoreactive bands that are correlated with increased CTSL expression as a function of cell culture time or that are reduced or eliminated by the presence of the CTSLi in the lysis buffer.

## Description

The cysteine protease cathepsin L (CTSL) has traditionally been thought to function in the general breakdown of proteins within endosomes and lysosomes. However, CTSL has also been found outside the cell where it acts on extracellular matrix proteins and other targets to contribute to diverse disease states, including cancer cell metastasis, cardiovascular and kidney diseases, and arthritis (Vidak et al. 2019). Early biochemical studies reported the presence of a CTSL-like protease activity in nuclear extracts that acted on a variety of transcription factors, including Rb, SP1, and retinoic acid receptor alpha (Nishinaka et al. 1997; Fu et al. 1998; Nagaya et al. 1998). A later study reported that a unique isoform of CTSL localized to the nucleus to regulate cell cycle progression via action on the CDP/Cux transcription factor (Goulet et al. 2004). Additional potential substrates of CTSL were later reported under specific cell culture conditions and cell types. For example, topoisomerase I was reported to become cleaved by CTSL in necrotic cells (Pacheco et al. 2005). Similarly, the core chromatin protein histone H3 has been reported to be cleaved by CTSL during embryonic stem cell differentiation (Duncan et al. 2008), oncogene-induced senescence (Duarte et al. 2014), and in the differentiated cells of mouse intestinal villi (Ferrari et al. 2021). Finally, expression of the DNA double-strand break repair protein 53BP1 was reported to be influenced by CTSL activity in cells lacking the nuclear structure protein lamin A (Gonzalez-Suarez et al. 2011), in cells starved of serum (Croke et al. 2013; Grotsky et al. 2013), during oncogene-induced senescence (Graziano et al. 2016), and in overconfluent cells (Mayca Pozo et al. 2017). In most of these reports, the cleavage of the substrate protein by CTSL was found to be correlated with increased expression of CTSL under the specific cell growth state.

In our ongoing studies of DNA damage responses in quiescent cells and human skin, we recently noted a unique band on immunoblots of the nucleotide excision repair protein XPA that was approximately 3-4 kDa smaller than the full-length protein (Khan et al. 2022). This band was not observed in sub-confluent cells or when quiescent cells were lysed using buffers that contained inhibitors of CTSL, including ionic detergents such as SDS (Krupa and Mort 2000; Pacheco et al. 2005), a commercial protease inhibitor cocktail containing the general cysteine protease inhibitors leupeptin and E-64, or the specific CTSL inhibitor Z-FY-CHO. Consistent with previous reports (Lockwood and Shier 1977; Zhang et al. 2017), we noted that CTSL protein levels greatly increased after cells reached full confluence and achieved a quiescent state characterized by the lack of further cell division. Because the cleavage of XPA only occurred *after* cells were lysed, we concluded that this cleavage product was a technical artifact of lysing cells with high CTSL levels in buffers lacking CTSL inhibitors.

As discussed above, several nuclear substrates have been reported for CTSL. However, several of these reports did not describe the use of CTSL or other protease inhibitors in the lysis buffers. We therefore examined whether some of these other purported functionally important CTSL substrates similarly become non-specifically cleaved during the lysis of quiescent cells. To explore this hypothesis, lysates were prepared from HaCaT keratinocytes at various time points after plating using a Triton X-100-based lysis buffer lacking inhibitors of CTSL or other proteases. As show in **Figure 1A** , the level of CTSL protein increased dramatically 3 days after plating (when cells reached full confluence) (Khan et al. 2022) and was correlated with the appearance of smaller immunoreactive, previously reported bands for multiple proteins, including XPA, 53BP1, and histone H3. Similar results were observed in U2OS osteosarcoma cells ( **Figure 1B** ) and NHF1 telomerase-immortalized neonatal foreskin fibroblasts ( **Figure 1C** ).

To determine whether the cleavage of these proteins occurred in the cell or during cell lysis, the lysis buffer was supplemented with either a general protease inhibitor cocktail containing the CTSL inhibitors E-64 and leupeptin, E-64 alone, the specific CTSL inhibitor Z-FY-CHO, or a cathepsin B inhibitor. As shown in **Figure 1D** , the cleaved bands for all three CTSL substrates was eliminated or greatly reduced by the presence of a CTSL inhibitor in the lysis buffer. Time course experiments in which the CTSL inhibitor Z-FY-CHO was added at various time points after resuspending quiescent cells in the lysis buffer showed a clear time-dependent increase in cleavage of these CTSL substrates ( **Figure 1E** ).

We were next interested in determining whether there may be other nuclear proteins that are acted upon by CTSL during the lysis of quiescent cells. We therefore performed protein immunoblotting with a panel of 21 different antibodies using lysates from quiescent HaCaT cells lysed in the absence or presence of the CTSL inhibitor Z-FY-CHO. As shown in **Figure 1F** , smaller immunoreactive bands consistent with protein cleavage were found for five additional proteins, including the DNA damage response protein kinases ATM and DNA-PK (catalytic subunit), MAT1 (a subunit of the DNA repair/transcription factor TFIIH), the 70-kDa subunit of the single-stranded DNA binding protein RPA, and the exonuclease TREX1. Thus, we conclude that many proteins can be cleaved by CTSL when cells expressing high levels of CTSL are lysed in the absence of CTSL inhibitors. It should be noted that the extent of cleavage varied from protein to protein, with XPA cleavage appearing to be a robust measure of CTSL activity in cell lysates.

CTSL has been reported to be elevated in a variety of cell growth states, including differentiation (Duncan et al. 2008; Ferrari et al. 2021), senescence (Duarte et al. 2014; Graziano et al. 2016), and quiescence (Croke et al. 2013; Grotsky et al. 2013; Mayca Pozo et al. 2017), and several studies of these studies did not describe the use of CTSL inhibitors during the preparation of cell lysates. Thus, to differentiate protein cleavage events that occur in the cell versus during cell lysis, we suggest that care should be taken to ensure that studies involving CTSL include the addition of active CTSL inhibitors during the preparation of cell or tissue lysates. Moreover, because the expression of other cathepsins and proteases may be elevated during quiescence and under other conditions of cell stress, our findings are likely relevant to other proteases and cellular proteins in addition to CTSL.

## Methods

HaCaT keratinocytes, U2OS osteosarcoma cells, and NHF1 fibroblasts were cultured in DMEM containing 10% fetal bovine serum, penicillin/streptomycin, and an additional 2 mM L-glutamine in a 37°C humidified 5% CO _2_ incubator. For time course experiments in Figure 1A-C, cells were plated in 6-well plates at a high enough density that they would be approximately 50% confluent one day after plating. Cells were harvested on the indicated days by aspiration of the culture media, scraping of the cells with 1 ml ice-cold PBS, a brief centrifugation to pellet the cells, and then aspiration of the remaining PBS. For all other experiments, cells were lysed 5 days after plating. The cell pellets were then either stored at -80°C or directly resuspended in lysis buffer. If frozen cell pellets were used, the cell pellets were quickly thawed for 10-15 sec in a 37°C water bath and then resuspended in Triton X-100 lysis buffer (20 mM Tris-HCl, pH 7.5, 150 mM NaCl, 1 mM EDTA, 1 mM EGTA, and 1% Triton X-100) and incubated on ice for 15-20 min with occasional vortexing. In some experiments, the lysis buffer was supplemented with either 1X HALT protease inhibitor cocktail (ThermoFisher 1860932), 30 µM E-64 (Sigma), 10 µM cathepsin L inhibitor II (Z-FY-CHO; Sigma 219426), or 10 µM of cathepsin B inhibitor CA074 (Tocris), as indicated. Soluble lysates were obtained by centrifugation at 20,000 x g for 15-20 min in a refrigerated microcentrifuge and added to tubes containing 1/6 volume of 6X SDS-PAGE sample buffer (50 mM Tris-HCl pH 6.8, 100 mM DTT, 5% glycerol, 1% SDS, and 0.005% bromophenol blue). The insoluble pellets were resuspended in 1X SDS-PAGE sample buffer and sonicated to solubilize histones. Lysates were separated SDS gels at 200 V using a Bio-Rad Mini-PROTEAN Tetra system. Proteins were transferred at 25 V for 12 min onto a 0.45 µm nitrocellulose membrane (Cytiva Amersham Protran 45-004-016) using a Bio-Rad Trans-Blot Turbo semi-dry transfer apparatus. Blots were then stained with 0.5% Ponceau S (Sigma P3504) with images of the stained blots obtained on a Molecular Imager Chemi-Doc XRS+ imaging system (Bio-Rad). The blots were blocked in 5% non-fat milk (Kroger) in TBST (Tris-buffered saline containing 0.1% Tween-20) and then probed overnight with 1:2000 to 1:5000 dilutions of the antibodies against the following proteins: XPA (Cell Signaling #14607), 53BP1 (Cell Signaling #4937), histone H3 (Cell Signaling #3638), CTSL (Cell Signaling #71298), ATM (Cell Signaling #2873), DNA-PK catalytic subunit (Cell Signaling #12311), MAT1 (Santa Cruz sc-6234), RPA70 (Bethyl A300-241), and Trex1 (Cell Signaling #15107). Following washing and incubation with HRP-conjugated secondary antibody (Invitrogen), chemiluminescence was visualized using Bio-Rad Clarity ECL reagent and Chemi-Doc XRS+ imaging system.

## References

[R1] Croke M, Neumann MA, Grotsky DA, Kreienkamp R, Yaddanapudi SC, Gonzalo S (2013). Differences in 53BP1 and BRCA1 regulation between cycling and non-cycling cells.. Cell Cycle.

[R2] Duarte LF, Young AR, Wang Z, Wu HA, Panda T, Kou Y, Kapoor A, Hasson D, Mills NR, Ma'ayan A, Narita M, Bernstein E (2014). Histone H3.3 and its proteolytically processed form drive a cellular senescence programme.. Nat Commun.

[R3] Duncan EM, Muratore-Schroeder TL, Cook RG, Garcia BA, Shabanowitz J, Hunt DF, Allis CD (2008). Cathepsin L proteolytically processes histone H3 during mouse embryonic stem cell differentiation.. Cell.

[R4] Ferrari KJ, Amato S, Noberini R, Toscani C, Fernández-Pérez D, Rossi A, Conforti P, Zanotti M, Bonaldi T, Tamburri S, Pasini D (2021). Intestinal differentiation involves cleavage of histone H3 N-terminal tails by multiple proteases.. Nucleic Acids Res.

[R5] Fu YH, Nishinaka T, Yokoyama K, Chiu R (1998). A retinoblastoma susceptibility gene product, RB, targeting protease is regulated through the cell cycle.. FEBS Lett.

[R6] Gonzalez-Suarez I, Redwood AB, Grotsky DA, Neumann MA, Cheng EH, Stewart CL, Dusso A, Gonzalo S (2011). A new pathway that regulates 53BP1 stability implicates cathepsin L and vitamin D in DNA repair.. EMBO J.

[R7] Goulet B, Baruch A, Moon NS, Poirier M, Sansregret LL, Erickson A, Bogyo M, Nepveu A (2004). A cathepsin L isoform that is devoid of a signal peptide localizes to the nucleus in S phase and processes the CDP/Cux transcription factor.. Mol Cell.

[R8] Graziano S, Johnston R, Deng O, Zhang J, Gonzalo S (2016). Vitamin D/vitamin D receptor axis regulates DNA repair during oncogene-induced senescence.. Oncogene.

[R9] Grotsky DA, Gonzalez-Suarez I, Novell A, Neumann MA, Yaddanapudi SC, Croke M, Martinez-Alonso M, Redwood AB, Ortega-Martinez S, Feng Z, Lerma E, Ramon y Cajal T, Zhang J, Matias-Guiu X, Dusso A, Gonzalo S (2013). BRCA1 loss activates cathepsin L-mediated degradation of 53BP1 in breast cancer cells.. J Cell Biol.

[R10] Khan S, Cvammen W, Anabtawi N, Choi JH, Kemp MG (2021). XPA is susceptible to proteolytic cleavage by cathepsin L during lysis of quiescent cells.. DNA Repair (Amst).

[R11] Krupa JC, Mort JS (2000). Optimization of detergents for the assay of cathepsins B, L, S, and K.. Anal Biochem.

[R12] Lockwood TD, Shier WT (1977). Regulation of acid proteases during growth, quiescence and starvation in normal and transformed cells.. Nature.

[R13] Mayca Pozo F, Tang J, Bonk KW, Keri RA, Yao X, Zhang Y (2017). Regulatory cross-talk determines the cellular levels of 53BP1 protein, a critical factor in DNA repair.. J Biol Chem.

[R14] Nagaya T, Murata Y, Yamaguchi S, Nomura Y, Ohmori S, Fujieda M, Katunuma N, Yen PM, Chin WW, Seo H (1998). Intracellular proteolytic cleavage of 9-cis-retinoic acid receptor alpha by cathepsin L-type protease is a potential mechanism for modulating thyroid hormone action.. J Biol Chem.

[R15] Nishinaka T, Fu YH, Chen LI, Yokoyama K, Chiu R (1997). A unique cathepsin-like protease isolated from CV-1 cells is involved in rapid degradation of retinoblastoma susceptibility gene product, RB, and transcription factor SP1.. Biochim Biophys Acta.

[R16] Pacheco FJ, Servin J, Dang D, Kim J, Molinaro C, Daniels T, Brown-Bryan TA, Imoto-Egami M, Casiano CA (2005). Involvement of lysosomal cathepsins in the cleavage of DNA topoisomerase I during necrotic cell death.. Arthritis Rheum.

[R17] Vidak E, Javoršek U, Vizovišek M, Turk B (2019). Cysteine Cathepsins and their Extracellular Roles: Shaping the Microenvironment.. Cells.

[R18] Zhang T, Wolfe C, Pierle A, Welle KA, Hryhorenko JR, Ghaemmaghami S (2017). Proteome-wide modulation of degradation dynamics in response to growth arrest.. Proc Natl Acad Sci U S A.

